# Phytochemical Profile and Chemopreventive Properties of Cooked Glutinous Purple Rice Extracts Using Cell-Based Assays and Rat Model

**DOI:** 10.3390/foods11152333

**Published:** 2022-08-04

**Authors:** Huina Guo, Arpamas Chariyakornkul, Warunyoo Phannasorn, Sugunya Mahatheeranont, Rawiwan Wongpoomchai

**Affiliations:** 1 Department of Biochemistry, Faculty of Medicine, Chiang Mai University, Chiang Mai 50200, Thailand; 2 School of Basic Medical Sciences, Youjiang Medical University for Nationalities, Baise 533000, China; 3 Functional Food Research Unit, Science and Technology Institute, Chiang Mai University, Chiang Mai 50200, Thailand; 4 Rice and Cereal Chemistry Research Laboratory, Department of Chemistry, Faculty of Science, Chiang Mai University, Chiang Mai 50200, Thailand

**Keywords:** antioxidant activity, antimutagenicity, anti-inflammatory activity, cooked rice, phytochemicals

## Abstract

Purple rice has gained attention for its health promoting potential due to a high content of bioactive phytochemicals. The heat generated during cooking alters the quality and quantity of nutrients and phytochemicals in food. This study aimed to investigate the phytochemical profile and chemopreventive properties of cooked glutinous purple rice using cell-based assays and a rat model. Purple rice was cooked in a rice cooker and was then further extracted with solvents to obtain dichloromethane and methanol extracts. The methanol extracts of glutinous purple rice contained great amounts of phenolics, flavonoids, and anthocyanins. Protocatechuic acid (2.26–5.40 mg/g extract) and cyanidin 3-glucoside (34.3–65.7 mg/g extract) were the major phenolic acid and anthocyanin contents, respectively. After cooking, the content of anthocyanins, γ-oryzanols, and phytosterols decreased, while the amount of some phenolic acid and tocol contents increased. Methanol extracts of glutinous purple rice inhibited reactive oxygen species production about 60% in PMA-treated peripheral blood mononuclear cells, reduced nitric oxide formation in LPS-induced RAW 264.7 cells (26–39% inhibition), and exhibited antimutagenicity against several mutagens using the Ames test, but dichloromethane extracts presented only mild anti-inflammatory activities. Although methanol extracts induced mild mutagenicity (mutagenic index 2.0–2.5), they did not induce micronucleated hepatocyte formation and certain hepatic CYP450 isozyme activities in rats. However, the mutagenicity of the methanol extract significantly declined after cooking. In summary, the methanol extract of the cooked glutinous purple rice might be a promising cancer chemopreventive fraction, which was neither genotoxic nor posing adverse effects on phytochemical–drug interaction in rats.

## 1. Introduction

Rice is one of the most commonly consumed foods worldwide, particularly throughout the Asian continent. Rice colors vary from white to brown, red, dark purple, and black [1]. Although about 85% of the rice consumed by humans is white [2], pigmented rice is being increasingly consumed due to its nutritional value and phytochemical constituents [3]. Purple rice, which contains high amounts of bioactive compounds and has exhibited chemopreventive efficacy, has gained attention for its health promoting potential [4]. The phytochemicals that exist in purple rice include phenolic compounds such as anthocyanins. These compounds have been found in higher quantities than in other varieties of colored rice [5,6,7,8]. Remarkably, hydrophobic compounds, such as ϒ-oryzanol and tocols, have been well characterized. Furthermore, *in vitro* and *in vivo* studies have reported that purple rice exhibits antioxidant, antimutagenic, anti-inflammatory, and anti-carcinogenic activities [9,10,11,12].

Even though a great deal of effort has been put forth in battling cancer, it is still one of the most difficult diseases to overcome. Late diagnosis, high costs, and minimally effective treatments for cancer have led to even greater amounts of attention being paid to developing preventive strategies for the disease. Cancer chemoprevention is well known for its ability to reduce incidences of cancer and to retard the formation of cancer. Chemo-preventive agents have been found to be capable of repairing DNA, inhibiting DNA mutations, quenching oxidative radicals, aiding in the detoxification of liver carcinogens, reducing organism inflammation, and maintaining the balance between proliferation and apoptosis. All of these can help in preventing the development of carcinogenesis, particularly in its early stages [13,14]. Rice is known to be a rich source of phytochemicals and to possess various biological activities, both of which make it a promising candidate in the development of effective cancer chemopreventive agents.

Typically, rice is consumed after it has been cooked, which can influence the nutritional make-up and final quality of the rice. Previous studies have shown that cooking generally causes a reduction in antioxidant capacity along with a reduction in phenolic contents in pigmented or non-pigmented rice. The extent of attenuation depends upon the rice variety and the cooking method [7,8,15,16]. Using a rice cooker and a water bath may retain more phenolic compounds compared to boiling, microwaving, and pressure cooker methods [7]. Considering the fact that rice cookers are so widely used in households throughout Asia, it was chosen to be used in this study. However, most studies have focused on the influence of the cooking method on certain phenolics and any important aspects of the biological activities of these phenolics, primarily with regard to the determination of their antioxidant activity using chemical assays such as ABTS, DPPH, and FRAP assays. A comprehensive understanding of biological activities of cooked purple rice evaluated using cell-based assays and animal models is still lacking. This study aimed to evaluate the phytochemical profile of various extracts obtained from purple glutinous rice during the household cooking process compared with uncooked rice. Furthermore, the relevant cancer chemopreventive properties, including antimutagenic, antioxidant, and anti-inflammatory activities, were investigated, and a safety assessment of the cooked rice extracts was conducted in cell culture system and rat model.

## 2. Materials and Methods

### 2.1. Chemicals

Aflatoxin B_1_ (AFB_1_), sodium azide (NaN_3_), phenylmethylsulfonyl fluoride (PMSF), resorufin, ethoxyresorufin, methoxyresorufin, erythromycin, phorbol 12-myristate 13-acetate (PMA), dihydrorhodamine 123 (DHR), and lipopolysaccharides (LPS) were obtained from Sigma Aldrich Corp (St. Louis, MO, USA). 2-Amino-3,4 dimethylimidazo [4,5-f] quinolone (MeIQ), 2-aminoanthracene (2-AA), and 2-(2-furyl)-3-(5-nitro-2-furyl)-acrylamide (AF-2) were provided by Wako Pure Chemicals (Osaka, Japan). Furthermore, 6-hydroxy-2,5,7,8-tetramethylchroman-2-carboxylic acid (Trolox) were acquired from Merck (Darmstadt, Germany). Collagenase type IV and 4′-6-diamidino-2-phenylindole (DAPI) were bought from the Gibco/Invitrogen Corp (Waltham, MA, USA). Fetal bovine serum (FBS), Dulbecco’s Modified Eagle Medium (DMEM) and Roswell Park Memorial Institute (RPMI) medium were purchased from Thermo Fisher Scientific Inc. (Waltham, MA, USA). All standard phytochemicals were of high-performance liquid chromatography (HPLC) grade, and all other chemicals were of analytical grade.

### 2.2. Preparation of Purple Rice Extracts

Glutinous purple rice grains (*Oryza sativa* L., PES 1 CMU) were provided by Dr. Sansanee Jamjod, Research Center for Development of Local Lanna Rice and Rice Products, Chiang Mai University, Thailand. The rice was cultivated from August to November of 2019. The herbarium number of this rice was CMU 023254. This rice was deposited at the Faculty of Pharmacy, Chiang Mai University, Chiang Mai, Thailand. Glutinous purple rice was cooked by a traditional method by 8 h soaking with distilled water (1:1.5, rice: water) at room temperature, and further processing in a rice cooker (MD, Thailand, 1.0 L) at approximately 100 °C for 40 min to obtain palatably cooked rice. After allowing the rice to cool down, the cooked rice was dehydrated using a lyophilizer. The dried cooked rice and raw rice were ground using an electric grinder (Tefal, DPA130, Thailand) before stepwise extraction in dichloromethane and methanol, as described elsewhere [12]. Briefly, after rice grains were macerated in dichloromethane for two days, the filtrates were evaporated and freeze-dried to obtain a dichloromethane extract of raw rice (DR) and a dichloromethane extract of cooked rice (DC). The resulting residues were further extracted with methanol for two days in order to obtain a methanol extract of the raw rice (MR) and a methanol extract of the cooked rice (MC) after administration of the filtration and drying processes.

### 2.3. Phytochemical Analysis

#### 2.3.1. Spectrophotometric Determination of Phenolic Compounds

The content of the total phenolic, flavonoid, and anthocyanin compounds of the extracts were evaluated using the Folin-Ciocalteu method, the aluminum chloride colorimetric method, and the pH-differential method, respectively [9]. Gallic acid and catechins were used as standards in the determination of total phenolics and flavonoids, respectively. Total anthocyanin content was calculated using the extinction coefficient of cyanidin-3-glucoside.

#### 2.3.2. HPLC Analysis of Phenolic Compounds and γ-Oryzanol

Phenolic acid, anthocyanin, and γ-oryzanol content was determined by using reverse phase HPLC, as has been described in our previous studies [17,18]. Ten microliters of each extract were injected into a reverse phase C18 column (4.6 mm × 250 mm, 5 μm) for determination of phenolic acids and anthocyanins using Zorbax Eclipse Plus C18 (Agilent Technologies, Santa Clara, CA, USA) and to measure the γ-oryzanol content using Inertsil ODS-3 (GL Sciences Inc., Tokyo, Japan). The phenolic acid content in the extracts was determined under conditions involving a gradient elution comprised of 3% acetic acid in deionized water and methanol as a mobile phase. The phenolic acid content was then measured at various wavelengths. Phenolic acid standards were composed of gallic acid, protocatechuic acid, 4-hydroxybenzoic acid, chlorogenic acid, vanillic acid, syringic acid, *p*-coumaric acid, ferulic acid and ellagic acid. The anthocyanin content was detected using a gradient mobile phase of 5% formic acid in deionized water and 5% formic acid in acetonitrile. The content was then measured at a wavelength of 520 nm. Anthocyanin standards included cyanidin, cyanidin-3-glucoside, delphinidin-3-glucoside, malvidin-3-glucoside, and peonidin-3-glucoside. The quantitative analysis of γ-oryzanol was performed using a diode array detector at a wavelength of 325 nm under the isocratic system of 35% acetonitrile in methanol. Accordingly, γ-oryzanol standards were composed of a mixture of cycloartenyl ferulate, 24-methylene cycloartanyl ferulate, campesteryl ferulate, and β-sitosteryl ferulate.

#### 2.3.3. Determination of Phytosterols and Tocols

Phytosterols and tocols were determined according to Phannasorn et al. (2021) [18]. For phytosterol measurements, five microliters of the samples were injected into a Kinetex PFP column (4.6 × 250 mm, 5 μm, Phenomenex, Inc., Torrance, CA, USA), and an Agilent HPLC 1100 was connected to a diode array detector (Model G1315 A; Agilent Technologies, Santa Clara, CA, USA). The mobile phase consisted of methanol and water at a constant flow rate of 1 mL/min for 30 min. Phytosterols were monitored at a wavelength 210 nm using a fluorescence detector (Model 1046A; Hewlett Packard, Palo Alto, CA, USA), while a normal phase silica column (VertiSep ^TM^ UPS 4.6 × 250 mm, 5 μm, Vertical Chromatography Co., Ltd., Nonthaburi, Thailand) was employed for tocol analysis. An isocratic elution of hexane:isopropanol:ethyl acetate:acetic acid at ratios of 97.6:0.8:0.8:0.8 v/v/v/v was established with a flow rate of 1 mL/min. Each form of tocol (α, β, γ, and δ forms of tocopherols and tocotrienols) were measured with the use of a fluorescence detector with an excitation value of 294 nm and an emission value of 326 nm.

#### 2.3.4. GC–MS Analysis of Hydrophobic Components

The hydrophobic phytochemical profiles, particularly of fatty acid derivatives and phytosterols of the purple rice extracts, was further analyzed using gas chromatography–mass spectrometry (GC–MS) (Clarus 690 gas chromatograph, PerkinElmer, Waltham, MA, USA). The GC column was employed an Elite-5MS column (30 m (L) × 0.25 mm (i.d.) × 0.25 μm film thickness, PerkinElmer, USA). The carrier gas was comprised of helium and administered at a constant flow rate of 1 mL/min. The GC oven temperature was conducted from 60 °C to 200 °C at a rate of 20 °C/min, from 200 °C to 280 °C at a rate of 10 °C/min, and was ultimately held for 22 min. One microliter of the sample was injected at an injector temperature of 250 °C with a split ratio of 20:1. The MS condition was carried out using a Clarus SQ8T mass spectrometry detector (PerkinElmer, USA). The ionization mode involved an electron impact ionization (EI) mode at 70 eV, and the ion source temperature was 230 °C. NIST libraries were used to identify the bioactive compounds by matching their mass spectra with the reference spectra obtained from the database.

### 2.4. Antioxidant Activity in Peripheral Blood Mononuclear Cells

The effects of the purple rice extracts on reactive oxygen species (ROS) production in peripheral blood mononuclear cells (PBMC) were evaluated. PMA was used as an ROS activator. The protocol was approved of by the Human Research Ethics Committee of the Faculty of Medicine, Chiang Mai University (8784/2022). Human peripheral blood obtained from healthy human volunteers was lyzed with hemolysis solution comprised of 155 µM NH_4_Cl, 12 µM NaHCO_3_, and 0.01 µM EDTA. The resulting leucocytes obtained from centrifugation were gently mixed with RPMI medium and placed onto two plates for the detection of cell viability and antioxidant activity. For the purposes of conducting a viability test, various concentrations of extracts ranging from 10 to 100 ug/mL were incubated with PBMC for 24 h in a CO_2_ incubator before 25 µg/mL of resazurin was added. After 2 h of interaction, the intensity of the fluorescent product was measured at excitation and emission wavelengths of 530 and 590 nm, respectively. In terms of the ROS production assay, cells were incubated with 1 µM DHR and stimulated with 1 µM PMA for 2 h. The fluorescence intensity of oxidized DHR by ROS was recorded every 5 min for 2 h, and the value was determined using 485 nm as the excitation wavelength and 520 nm as the emission wavelength [19]. Trolox was used as an antioxidant reference.

### 2.5. Anti-Inflammatory Activity in Murine Macrophage Cells

Murine macrophage RAW 264.7 cells (TIB-71TM, ATCC) purchased from American Type Culture Collection were cultured in DMEM containing 10% fetal bovine serum and 1% penicillin/streptomycin. The non-toxic concentrations of glutinous purple rice extracts were checked using the MTT assay. RAW 264.7 cells (2 × 10^4^ cells/well) were treated with various concentrations of rice extracts ranging from 0 to 300 μg/mL for 2 h and then incubated for 24 h in the absence or presence of 1 μg/mL LPS. Griess reagent containing naphthylethylenediamine dihydrochloride in water and sulfanilamide in phosphoric acid was added to determine nitric oxide production [20]. The absorbance of the azo dye product was measured at a wavelength of 550 nm and compared with the standard curve of sodium nitrite.

### 2.6. Mutagenic and Antimutagenic Activities in Salmonella typhimurium

The *Salmonella* mutation assay was used to determine the mutagenicity and antimutagenicity of the glutinous purple rice extracts. *Salmonella typhimurium* strains TA98 and TA100, kindly provided by Dr. Kei-Ichi Sugiyama, National Institute of Health, Tokyo, Japan, were used. For the mutagenicity test, a vehicle control, a test extract or a standard mutagen was mixed with S9 mix or phosphate buffer, pH of 7.4, and preincubated with bacterial culture at 37 °C for 20 min. AF-2 and 2-AA served as positive controls in the absence and presence of metabolic activation, respectively. After preincubation, the top agar containing 0.5 mM L-histidine/D-biotin was added to the mixture, which was then poured onto the minimal agar plate and further incubated for 48 h. A test substance was considered a possible mutagen when the number of revertant colonies was two-fold higher than the number of spontaneous revertant colonies in the vehicle control [21].

In terms of the antimutagenicity of glutinous purple rice extracts, various environmental mutagens, including AF2 and AFB_1_ for TA98, and NaN_3_ and MeIQ for TA100, were used to induce mutagenesis. The percentage of inhibition was calculated as has been described previously [21]. Vanillic acid was used as a positive antimutagen [21].

### 2.7. Clastogenic Activity in Rats

The clastogenicity of some of the purple rice extracts, the mutagenic index of which was more than 2.0, was determined by administering the rat liver micronucleus test. Male Wistar rats (5-week-olds) weighing between 120 and 130 g were obtained from Nomura Siam International, Thailand. Rats were housed in the Laboratory Animal Center, Chiang Mai University under the following controlled conditions: a temperature of 25 ± 1 °C, a 12-h light-dark cycle, free access to water, and a standard diet. All rats were adapted to the environment a week before initiating the experiment. The experimental protocol was approved of by the Animal Ethics Committee, Chiang Mai University (01/2565). To investigate the *in vivo* genotoxicity of the methanol extracts of glutinous purple rice, rats were randomly divided into three groups of 6 rats per group. Group 1 served as a vehicle control, while rats in groups 2 and 3 were intragastrically fed with methanol extracts of both raw rice and cooked rice, respectively, at a dose of 1000 mg/kg BW for 14 days. The experimental protocol accorded with ICH M3(R2) guideline with some modification [22]. On day 15, rats were anesthetized by 2% isoflurane with oxygen inhalation before performing a partial hepatectomy to induce hepatic cell growth. Four days after the surgery, rats were euthanized by intraperitoneal injection of 70 mg/kg BW of thiopental sodium, and their hepatocytes were isolated using the 2-step collagenase liver perfusion method. The obtained hepatocyte suspension was stained with an equal volume of 4′,6-diamidino-2-phenylindole. The number of micronucleated hepatocytes, binucleated cells, and mitotic cells were counted under a fluorescent microscope (AX-70, Olympus, Tokyo, Japan) based on the method described in previous studies [21].

### 2.8. Determination of Xenobiotic Metabolizing Enzyme Activities in Rat Livers

Xenobiotic metabolizing enzyme activities were determined in the microsome of livers by ultracentrifugation [18]. The amount of microsomal protein was determined using the Lowry method. The activities of CYP1A1 and 1A2 were analyzed using the ethoxyresorufin-*O*-deethylation and methoxyresorufin-*O*-demethylation methods, respectively [23]. Ethoxyresorufin or methoxyresorufin, a substrate of CYP1A1 or 1A2, respectively, was mixed with the microsomal fraction of each rat under the presence of NADPH. The formation of the resorufin product was measured at an excitation wavelength of 520 nm and an emission wavelength of 590 nm. The obtained value was calculated from the standard curve of resorufin. CYP 3A activity was determined by employing the erythromycin *N*-demethylation method. Erythromycin was demethylated by CYP3A to produce formaldehyde, which was then absorbed at a wavelength of 405 nm and compared with the calibration curve of formaldehyde [23].

### 2.9. Statistical Analysis

All results were expressed as mean ± SD values with the exception of the results obtained from the *Salmonella* mutation assay, which were expressed as mean ± SEM values. The statistics analysis was performed by SPSS Software version 22 (SPSS Inc., Chicago, IL, USA). The significance of the differences between 2 groups was calculated by Student’s *t*-test, and more than 2 groups was analyzed by one-way ANOVA followed by administering Bonferroni post hoc tests. Data were verified for normality using a Kruskal–Wallis test. Accordingly, any differences were determined as significant when *p* < 0.05.

## 3. Results

### 3.1. Phytochemical Constituents in Glutinous Purple Rice

To determine the hydrophilic and low polar phytochemicals in cooked and uncooked glutinous rice, methanol and dichloromethane were used in this study. MC and MR contained greater amounts of total phenolic, flavonoid, and anthocyanin compounds than DC and DR when detected by spectrophotometry (Table 1). Using HPLC analysis, protocatechuic acid was the prominent phenolic acid, while cyanidin 3-glucoside was the major anthocyanin in MC and MR (Table 1). Furthermore, all hydrophilic phenolic compounds were significantly reduced after the cooking process. Notably, the contents of certain anthocyanins, including cyanidin 3-glucoside, peonidin 3-glucoside, and delphinidin 3-glucoside were reduced in MC. However, the amounts of protocatechuic acid and vanillic acid in MC increased when compared to MR (Table 1). The total phenolic, flavonoid, and anthocyanin compounds were not detected in DR and DC (data not shown).

The total contents of γ-oryzanols and phytosterols in DR were higher than in MR; however, the total amount of tocols in MR was higher than in DR (Table 2). Accordingly, γ-tocotrienol was the major form of tocol present in the glutinous purple rice. After being cooked, the contents of total γ-oryzanols and phytosterols were decreased. The γ-oryzanol and phytosterol contents in the dichloromethane extract were decreased but were increased in the methanol extract when compared with the uncooked extracts. Interestingly, the total content of tocols in DC and MC increased after the rice was cooked in a rice cooker (Table 2). All detected isoforms of tocols, with the exception of δ-tocopherol, were increased. Remarkably, MR and MC contained both hydrophobic and hydrophilic phytochemicals.

The identified phytochemicals in glutinous purple rice extracts analyzed by GC–MS are presented in Table 3. The identified compounds in the cooked rice extracts were greater than those of the raw rice extracts. The major components found in the glutinous purple rice extracts were fatty acid derivatives and phytosterols. The main identified compounds in the raw rice extracts were β-sitosterol, 1,19-eicosadiene, 9,12-octadecadienoic acid (linoleic acid), and hexadecanoic acid (palmitic acid), which were similar to the main identified compounds in the cooked rice extracts.

### 3.2. Antioxidant Activity of Glutinous Purple Rice Extracts

**Figure 1:**
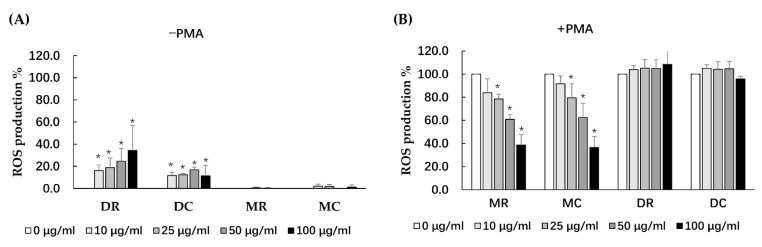
Effects of various extracts of glutinous purple rice on ROS production in peripheral blood mononuclear cells under conditions of: (**A**) an absence of PMA (−PMA), and (**B**) the presence of PMA (+PMA). Accordingly, *: significantly different than the negative control, *p* < 0.05. MR: methanol extract of raw rice, MC: methanol extract of cooked rice, DR: dichloromethane extract of raw rice and DC: dichloromethane extract of cooked rice.

The antioxidant extracts of glutinous purple rice were further investigated for their potential cellular defenses against ROS production in peripheral blood mononuclear cells (PBMC). All extracts of the glutinous purple rice at concentrations ranging from 0 to 100 μg/mL were non-toxic to PBMC viability (data not shown). All extracts did not induce ROS production in PBMC with the exception of dichloromethane extracts, which presented a mild degree of induction (Figure 1A). Moreover, MR and MC, but not DC and DR, inhibited ROS production in PBMC treated by PMA in a dose-dependent manner (Figure 1B). Notably, the inhibitory ROS capacity of the methanol extracts of cooked and uncooked purple rice was not determined to be different. ROS production induced by PMA was approximately 40% by the methanol extracts of purple rice at 100 μg/mL, while ROS production was equal to 46.8% by Trolox at 100 μM.

### 3.3. Anti-inflammatory Activity of Glutinous Purple Rice Extracts

**Figure 2:**
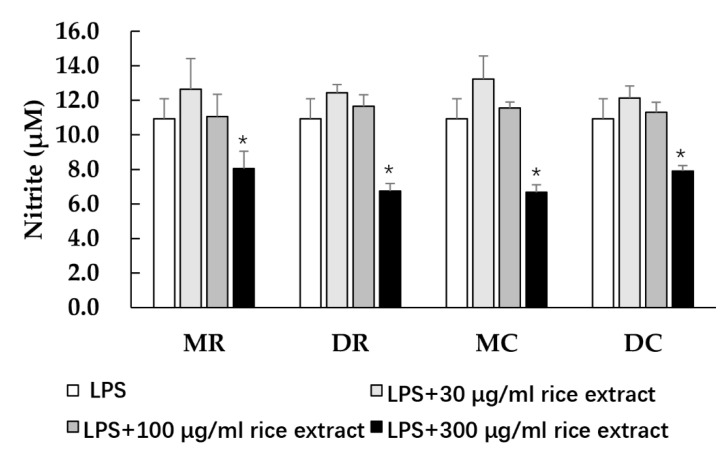
The effects of purple rice extracts on lipopolysaccharide (LPS)-induced nitric oxide production. Accordingly, *: significantly different than the negative control, *p* < 0.05. MR: methanol extract of raw rice, MC: methanol extract of cooked rice, DR: dichloromethane extract of raw rice, and DC: dichloromethane extract of cooked rice.

NO can be produced in macrophages when attacked by some endotoxins, such as LPS, leading to cellular inflammatory responses. The effects of glutinous purple rice on NO production were observed in RAW 264.7 cells. The various glutinous purple rice extracts at ranges from 0 to 500 μg/mL did not affect the cell viability of macrophages (data not shown). All rice extracts at high concentrations significantly lowered the NO levels in LPS-treated macrophages. (Figure 2). No significant differences in the NO inhibitory effects among the methanol and dichloromethane extracts were observed. Remarkably, the anti-inflammatory activity of rice after being cooked still endured.

### 3.4. Mutagenicity and Antimutagenicity of Glutinous Purple Rice Extracts

**Figure 3:**
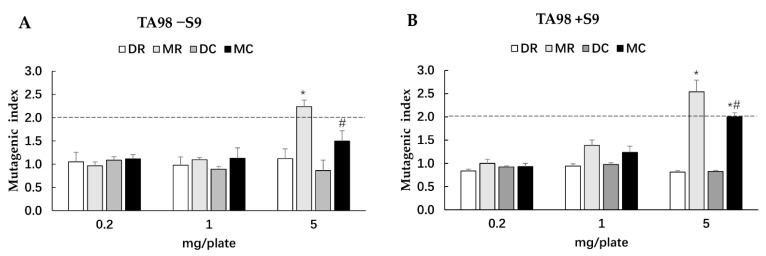
Mutagenicity of various extracts of glutinous purple rice in *S. typhimurium* strain TA98 under the following conditions: (**A**) an absence of S9 (TA98−S9), and (**B**) the presence of S9 (TA98 +S9). MR: methanol extract of raw rice, MC: methanol extract of cooked rice, DR: dichloromethane extract of raw rice, DC: dichloromethane extract of cooked rice. *: significantly different than the control (mutagenic index is 1.0), *p* < 0.05; #: significantly different than MR.

**Figure 4:**
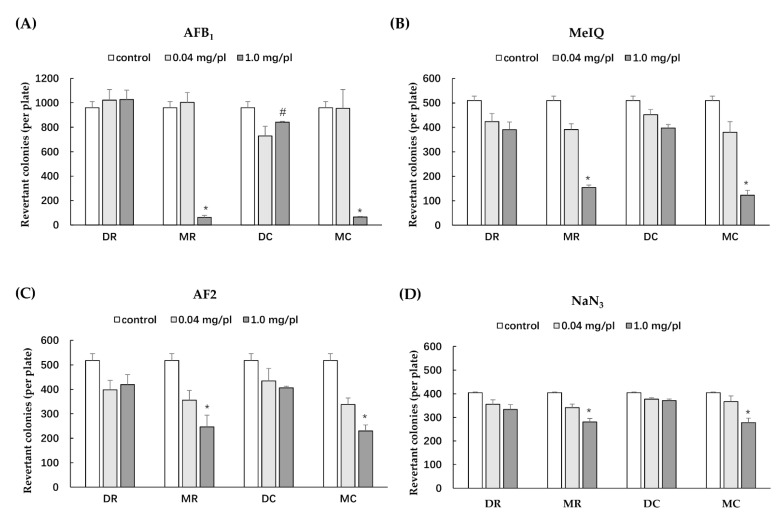
Antimutagenicity of various extracts of glutinous purple rice in *S. typhimurium* strains TA98 and TA100 against mutagenesis induced by: (**A**) AFB_1_ (TA98), (**B**) MeIQ (TA100), (**C**) AF-2 (TA98), and (**D**) NaN_3_ (TA100). MR: methanol extract of raw rice, MC: methanol extract of cooked rice, DR: dichloromethane extract of raw rice, and DC: dichloromethane extract of cooked rice. *: significantly different than the control, *p* < 0.05. #: significantly different than DR, *p* < 0.05.

The *Salmonella* mutation assay has been widely used to screen for the potential mutagenicity and antimutagenicity effects of a range of substances including extracts. The highest concentration of all glutinous purple rice extracts (5 mg/plate) showed no toxicity in both strains TA98 and TA100 under the presence and absence of metabolic activation (data not shown). All extracts did not reveal any mutagenicity in the TA100 strain. Moreover, dichloromethane extracts did not display any degree of mutagenicity in TA98, while the methanol extracts of glutinous purple rice extracts presented a mutagenic index of over 2.0, suggesting mutagenicity (Figure 3). Notably, the mutagenicity of the methanol extract significantly declined after cooking. Furthermore, MR and MC significantly inhibited the mutagenesis induced by indirect mutagens, namely AFB_1_ and MeIQ. Vanillic acid, a known antimutagen and anticarcinogen in rice [21,24], at 2 mg/plate presented 65.8% inhibition, while MR and MC at 1 mg/plate displayed 86.5% and 93.7% inhibition, respectively, against MeIQ-induced mutagenesis in the TA100 strain. Moreover, MR and MC could effectively inhibit AFB_1_ mutagenicity in the TA98 strain by approximately 96%, whereas vanillic acid could reduce mutagenicity by only 76.6%. Furthermore, MR and MC mildly reduced mutagenesis induced by direct mutagens, namely, AF-2 and NaN_3_, in a dose-dependent manner (Figure 4). However, no significant differences were observed in the antimutagenic capacity between MR and MC. DR and DC did not produce an antimutagenic effect on various mutagens. These results implied that some phytochemicals present in the methanol extracts of glutinous purple rice were antimutagenic and persisted after being cooked.

### 3.5. Clastogenicity and Cytochrome P450 Activities of Methanol Extracts of Glutinous Purple Rice in Rats

The rat liver micronucleus test was used to prove the mutagenicity of the methanol extracts of glutinous purple rice detected by the *Salmonella* mutation assay. A micronucleus was formed when the chromosomes were not incorporated into daughter cells after cell division [25]. The administration of MC or MR at 1000 mg/kg BW for 14 days did not affect the number of micronucleated cells, binucleated cells, and the mitotic index, indicating non-genotoxicity (Table 4). Furthermore, both MR and MC did not alter the activities of cytochrome P4501A1, 1A2, and 3A4 in rat livers.

## 4. Discussion

Cooking is an important food preparation method that reduces microbial growth and some antinutrient factors in food. The heat produced during the cooking process has been known to destroy certain nutrients in food, such as vitamin C and vitamin B, resulting in a reduction in the nutritional value of the food [26,27]. Chatthongpisut and her colleagues reported that cooking could reduce the content of total phenolic and anthocyanin compounds, along with the radical scavenging activities, in Thai purple rice [28]. Our study found that the household cooking process of purple glutinous rice using an electric rice cooker altered some beneficial phytochemicals, while its biological functions were retained.

Colored rice is a great source of beneficial phytochemicals. Accordingly, solvent polarity is a significant concern when determining either the phytochemical content or the relevant biological and pharmacological activities of rice. While the whole phenolic contents, particularly those of certain anthocyanins, such as cyanidin-3-glucoside, peonidin-3-glucoside and delphinidin-3-glucoside, in cooked purple glutinous rice were degraded, some phenolic acid contents, including protocatechuic aid and vanillic acid, were increased. This observation was in line with the reported outcomes of several studies on the alteration of phenolic compounds during cooking. The content of anthocyanin 3-glycoside in colored rice obtained by a pressure cooker was lower than that of cooked rice processed by a rice cooker and a gas range. Nevertheless, the increase of protocatechuic acid content was observed in these cooking methods [29]. Under thermal conditions, major anthocyanins can be degraded to anthocyanidin aglycones and produce several phenolic acids upon their structures. A- and B-rings of cyanidin are likely unstable at neutral conditions, which results in them being further transformed to phloroglucinaldehyde and subsequently protocatechuic acid [30]. In these cases, peonidin and delphinidin could be cleaved to become vanillic acid and gallic acid, respectively. For these reasons, the amounts of protocatechuic acid and vanillic acid were related to cyanidin-3-glucoside and peodinin-3-glucoside [31]. Gallic acid could not be detected in this study due to the small content of delphinidin.

Low polar constituents in rice, such as γ-oryzanols, phytosterols, and tocols, which are mainly found in bran, play various vital protective roles in offering protection against several degenerative diseases [32]. Some interesting results indicated that both the γ-oryzanol and phytosterol contents of cooked glutinous purple rice eluted by dichloromethane were decreased, but those that were produced by methanol were increased. It was therefore suggested that the increased γ-oryzanol content in cooked rice might be due to the release of some bound γ-oryzanol molecules, while it could be decreased by oxidative removal [33]. The alterations of phytosterols in glutinous purple rice after being cooked exhibited the same pattern that was found in γ-oryzanols. Phytosterols occur naturally in the esterified form with fatty acids in cell membranes [34]. The heat produced during cooking may release them into free forms within the cells, while some of them undergo further oxidation. Furthermore, the GC–MS analysis of glutinous purple rice supports the contention that the cooking process produces more kinds of low polar phytochemicals, including phytosterols, when compared with raw rice.

Tocopherols and tocotrienols are mainly attributed as a complex between tocols and phospholipids, while their link could be effectively broken during the heating process [35,36]. This may account for why the tocol contents in glutinous purple rice increased after the rice was cooked. Another study also reported that parboiled rice contained higher amounts of certain lipophilic antioxidants, such as tocols, when compared to uncooked brown rice [37]. Interestingly, the amount of tocols in the methanol extracts was greater than that in the dichloromethane extract. Previous studies have suggested that the hydroxyl groups on the chroman rings of tocols tended to dissolve and were more extractable in alcohol than their lower polar solvents [38]. Overall, the alteration of phytochemical profiles in glutinous purple rice after being cooked with an electric rice cooker might be caused by the release of phytochemicals from linked macromolecules or conjugated biomolecules in the cellular matrix or may occur from the degradation or oxidation of either their parent or intermediate compounds.

Carcinogenesis has been known to be mostly involved with DNA mutation, oxidative stress, and inflammation, leading to dysregulation of cellular homeostasis [39]. Certain phytochemicals in food that play a role in cancer prevention principally act as antimutagens, antioxidants, and anti-inflammatory agents to either ameliorate or inhibit the development of carcinogenesis [40]. Oxidative stress is related to the disruption of the balance between free radical production and the antioxidant defense system. The hydroxyl groups of the phenolic compounds are known to rapidly quench hydroxyl radicals by donating hydrogen atoms or electrons [41].The methanol extracts of both the cooked and uncooked purple rice presented great inhibitory effects on ROS production induced by PMA, a cytoplasmic NADPH oxidase activator in murine macrophages. It was implied that phenolic compounds may be representative of the antioxidant phytochemicals in glutinous purple rice. Furthermore, there are other mechanisms involved in the ROS defense pathways within the cellular system, such as the antioxidant enzyme system [42].

NO is one of the proinflammatory mediators that induce inflammation. Excessive production of NO under various pathological conditions can interact with either free radicals or biomolecules, leading to the promotion of certain degenerative diseases including cancer [43]. Interestingly, both of the methanol and dichloromethane extracts of cooked purple rice suppressed NO production induced by LPS when compared to the raw rice extracts. It can be suggested that hydrophilic and low polar compounds, which were heat-stable in glutinous purple rice, exhibited anti-inflammatory activities in murine macrophages. This result was in line with other findings that cooking black rice using a rice cooker still retains the inhibitory effects of the rice in terms of the production of NO, tumor necrosis factor-α, and interleukin-6 in LPS-induced macrophages [20]. Furthermore, δ-tocotrienol and the anthocyanins isolated from rice bran could inhibit the NO formation caused by the induction of some pro-inflammatory cytokines in LPS-activated macrophages [44,45].

DNA mutation can cause genotoxicity, which is one of important factors that initiates cancer formation [46]. The *Salmonella* mutation assay involves measuring the reverse mutations in several constructed *Salmonella* strains that are known to carry a particular histidine operon mutation [47]. The methanol extracts of glutinous purple rice, which contained high contents of phenolic compounds and tocols, exhibited antimutagenicity against several environmental mutagens, particularly AFB_1_ and MeIQ found in food products and grilled foods, respectively. The cooking process did not reduce the antimutagenicity of purple rice. Notably, the methanol extracts of purple rice at high doses presented mild mutagenicity in the bacterial TA98 strain. These results were similar to those of our previous studies, which indicated that the acidified methanol extract of purple rice hulls exhibited both mutagenicity and antimutagenicity in the *Salmonella* mutation assay [10]. Several studies have reported that some structures of flavonoids containing the free hydroxyl group at position 3, the double bond linking positions 2 and 3, and a keto group at position 4 comprised of quercetin and kaempferol displayed mutagenicity in the bacterial mutation test [48,49] but were not carcinogenic to animals and humans [50,51]. However, the methanol extract of cooked purple rice did not induce the formation of the micronuclei that were produced when chromosomes were not incorporated into the daughter cells after cell division, suggesting no clastogenicity [25]. Furthermore, the methanol extracts did not affect the xenobiotic enzyme activities of CYP1A1, CYP1A2, and CYP3A2. CYP3A4 is the main CYP450 enzyme responsible for drug metabolism, while the CYP1A family takes part in toxicant metabolism, such as aflatoxin B1 and pyrolysated meat products [52]. It can be suggested that the methanol extracts of glutinous purple rice did not produce any adverse effects on phytochemical–drug interactions.

## 5. Conclusions

Cooked glutinous purple rice extracts presented antioxidant, anti-inflammatory, and antimutagenic activities using a cell-based system. The hydrophilic compounds containing phenolic acids, flavonoids, and anthocyanins in a methanol extract exhibited antioxidant and antimutagenic activities, while the low polar compounds including γ-oryzanol, phytosterols, and tocols in methanol and dichloromethane extracts played a role in anti-inflammatory activities. The methanol extract of the cooked glutinous purple rice was the promising cancer chemopreventive fraction, which was neither genotoxic nor posing adverse effects on phytochemical–drug interactions in rats. The outcomes of this study have provided a more comprehensive perception of cooked glutinous purple rice as a potential source of cancer chemopreventive agents. Further studies are needed to obtain additional evidence on its effect on chemopreventive properties using animal models and clinical trials.

## Figures and Tables

**Table 1:** The content of phenolic phytochemicals in methanol extracts of glutinous purple rice.

Compounds	Methanol Extract of Cooked Rice	Methanol Extract of Raw Rice
Spectrophotometry (per g extract)		
Total phenolics (mg GAE)	132 ± 4.98 *	189 ± 11.4
Total flavonoids (mg CE)	101 ± 12.4 *	137 ± 10.7
Total anthocyanins (mg)	4.46 ± 0.55 *	8.22 ± 0.03
HPLC (mg per g extract)		
Protocatechuic acid	5.40 ± 0.02 *	2.26 ± 0.00
Vanillic acid	1.18 ± 0.00 *	0.75 ± 0.00
Delphinidin-3-glucoside	ND	3.73 ± 0.10
Cyanidin-3-glucoside	34.3 ± 0.50 *	65.7 ± 0.78
Peonidin-3-glucoside	5.53 ± 0.59 *	13.7 ± 0.31

Values are expressed as mean ± SD values. ND: not detected, GAE: gallic acid equivalent, CE: catechin equivalent, *: significantly different than the methanol extract of raw rice (*p* < 0.05).

**Table 2:** The contents of low-polar phytochemicals in glutinous purple rice extracts.

Compounds (Per Gram Extract)	Raw Rice		Cooked Rice	
	DCM	Methanol	DCM	Methanol
Gamma-oryzanol (mg)	194 ± 0.74	8.82 ± 0.21	67.5 ± 0.03 *	27.4 ± 0.29 ^#^
Cycloartenyl ferulate (mg)	59.9 ± 0.07	2.12 ± 0.06	15.4 ± 0.10	6.52 ± 0.18
24-methylene cycloartanyl ferulate (mg)	41.9 ± 0.20	1.23 ± 0.04	8.12 ± 0.02	3.31 ± 0.05
Campesteryl ferulate (mg)	28.3 ± 0.72	2.36 ± 0.28	15.7 ± 0.01	6.20 ± 0.06
β-Sitosteryl ferulate (mg)	63.4 ± 0.25	3.10 ± 0.16	28.3 ± 0.13	11.4 ± 0.12
Total Phytosterols (μg)	1735 ± 2.91	1337± 1.30	867 ± 7.62 *	1573 ± 16.8 ^#^
Stigmasterol + campesterol (μg)	983 ± 4.02	519 ± 3.41	391 ± 8.78	573 ± 9.13
β-sitosterol (μg)	752 ± 2.35	818 ± 4.16	476 ± 4.61	1000 ± 8.70
Total tocols (μg)	156 ± 11.4	202 ± 3.75	283 ± 4.93 *	303 ± 0.89 ^#^
α-tocopherol (μg)	12.8 ± 0.86	10.1 ± 1.23	25.0 ± 1.17 *	27.2 ± 0.76 ^#^
β-tocopherol (μg)	8.61 ± 0.85	12.0 ± 0.51	9.28 ± 0.83 *	17.4 ± 1.17 ^#^
γ-tocopherol (μg)	31.3 ± 2.41	14.7 ± 1.82	42.6 ± 2.05 *	35.9 ± 2.50 ^#^
δ-tocopherol (μg)	3.79 ± 0.11	13.2 ± 0.97	2.13 ± 0.24	5.06 ± 0.51
α-tocotrienol (μg)	4.11 ± 0.05	11.6 ± 0.32	11.7 ± 0.40 *	17.8 ± 0.50 ^#^
γ-tocotrienol (μg)	86.4 ± 6.96	123 ± 2.08	181 ± 1.50 *	183 ± 0.40 ^#^
δ-tocotrienol (μg)	8.97 ± 0.42	16.6 ± 0.65	10.4 ± 0.43 *	16.9 ± 0.49 ^#^

Values are expressed as mean ± SD values. DCM: dichloromethane, *: significantly different than the DCM extract of raw rice (*p* < 0.05), and ^#^: significantly different than the methanol extract of raw rice (*p* < 0.05).

**Table 3:** The identified compounds obtained from various extracts of glutinous purple rice using GC–MS analysis.

Retention Time (min)	Identified Compound	Dichloromethane Extract				Methanol Extract			
		Raw Rice		Cooked Rice		Raw Rice		Cooked Rice	
		Relative Content (%)	Quality (%)	Relative Content (%)	Quality (%)	Relative Content (%)	Quality (%)	Relative Content (%)	Quality (%)
5.45	8-methyl-1-decene	1.26	91.1	1.22	89.2	-		-	
5.53	hexadecane	-		0.16	84.2	-		-	
8.24	heptacosane	-		0.69	86.6	-		-	
10.01	hexadecanoic acid	3.22	94.7	2.92	91.0	13.1	97.6	4.80	98.0
11.34	9,12-octadecadienoic acid	9.74	95.7	10.7	93.0	34.8	96.1	16.6	97.4
11.38	1,19-eicosadiene	23.7	93.9	16.9	94.7	29.3	92.5	16.2	92.4
14.21	hexadecanoic acid, 2-hydroxy-1-(hydroxymethyl)ethyl ester	-		-		4.17	88.2	3.30	90.9
15.55	z,e-2-methyl-3,13-octadecadien-1-ol	-		-		4.84	84.8	10.4	91.4
16.63	squalene	-		3.12	85.6	-		1.93	94.0
22.78	campesterol	-		-		-		4.15	84.4
23.37	stigmasterol	7.75	85.1	4.31	86.5	-		10.5	92.0
24.78	β-sitosterol	38.5	87.6	35.5	86.7	12.8	83.5	24.1	81.3
26.97	cycloartenol acetate	2.62	80.9	7.83	85.6	-		2.95	84.5
	Total identification (%)	86.8		83.3		98.9		94.9	

**Table 4:** Effects of the 14-day administration of methanol extracts of raw and cooked rice on clastogenicity and the activity of some cytochrome P450 isozymes in rat livers.

Parameters	5% Tween 80	Methanol Extract	
		Raw Rice	Cooked Rice
Micronucleated cells (/1000 hepatocytes)	1.40 ± 0.66	0.83 ± 0.40	1.08 ± 0.73
Binucleated cells (/1000 hepatocytes)	7.81 ± 1.90	6.38 ± 0.74	5.90 ± 1.56
Mitotic index (%)	0.25 ± 0.60	0.00 ± 0.00	0.17 ± 0.26
CYP1A1 activity (fmole/min/mg/protein)	1.53 ± 0.91	1.56 ± 0.26	1.89 ± 1.20
CYP1A2 activity (fmole/min/mg/protein)	2.23 ± 1.32	2.07 ± 0.42	2.31 ± 1.02
CYP3A2 activity (pmole/min/mg/protein)	4.87 ± 1.15	3.74 ± 0.63	4.98 ± 1.14

Values are expressed as mean ± SD values. CYP: cytochrome P450.

## Data Availability

The data presented in this study are available upon request from the corresponding author.
